# Working title: high dose rate intra-cavitary brachytherapy with cobalt 60 source for locally advanced cervical cancer: the Zimbabwean experience

**DOI:** 10.1186/s13027-020-00340-5

**Published:** 2021-01-07

**Authors:** Shirley Chibonda, Ntokozo Ndlovu, Nomsa Tsikai, Lameck Munangaidzwa, Sandra Ndarukwa, Albert Nyamhunga, Tinashe Mazhindu

**Affiliations:** 1 Parirenyatwa Hospital Radiotherapy and Oncology Centre, Harare, Zimbabwe; 2 Department of Oncology, University of Zimbabwe Faculty of Medicine and Health Sciences, Harare, Zimbabwe; 3 Department of Statistics, National AIDS Council of Zimbabwe, Harare, Zimbabwe; 4 Department of Oncology, Sally Mugabe Central Hospital, Harare, Zimbabwe

## Abstract

**Background and purpose:**

Cervical cancer is the fourth commonest cancer in women in the world with the highest regional incidence and mortality seen in Southern, Eastern and Western Africa. It is the commonest cause of cancer morbidity and mortality among Zimbabwean women. Most patients present with locally advanced disease that is no longer amenable to surgery. Definitive concurrent chemoradiation (CCRT), which is the use of external beam radiotherapy (EBRT) and weekly cisplatin, includes use of intracavitary brachytherapy, as the standard treatment. In the setting of this study, cobalt-60 (Co^60^)-based high dose rate brachytherapy (HDR-BT) has been in use since 2013. This study sought to review practices pertaining to use of brachytherapy in Zimbabwe, including timing with external beam radiotherapy, adverse effects and patient outcomes.

**Methods:**

A retrospective analysis of data from records of patients with histologically confirmed cervical cancer treated with HDR-BT at the main radiotherapy centre in Zimbabwe from January 2013 to December 2014 was done. Outcome measures were local control, overall survival as well as gastro-intestinal and genito-urinary toxicity.

**Results:**

A total of 226 patients were treated with HDR-BT during the study period, with a 97% treatment completion rate. All patients received between 45-50Gy of pelvic EBRT. Seventy-four percent received concurrent platinum-based chemotherapy. In 52% of the patients, HDR-BT was started when they were still receiving EBRT. The commonest fractionation schedule used was the 7Gy × 3 fractions, once a week (87%). Clinical complete tumour response was achieved in 75% at 6 weeks post treatment, 23% had partial response. Follow-up rates at 1 year and 2 years were 40 and 19% respectively. Disease free survival at 1 year and 2 years was 94 and 95% respectively. Vaginal stenosis was the commonest toxicity recorded, high incidence noted with increasing age. Four patients developed vesico-vaginal fistulae and two patients had rectovaginal fistulae.

**Conclusion:**

One hundred and seventeen patients patients started HDR-BT during EBRT course, with a treatment completion rate of 97%. The overall treatment duration was within 56 days in the majority of patients. Early local tumour control was similar for all the HDR-BT fractionation regimes used in the study, with a high rate (75%) of complete clinical response at 6 weeks post-treatment. Prospective studies to evaluate early and long-term outcomes of HDR-BT in our setting are recommended.

## Introduction

Cervical cancer is the 4th most common cancer worldwide. It remains the most common cancer in women living in low and middle income countries (LMICs) [1]. In developed countries, morbidity and mortality from this malignancy have decreased due to effective screening programmes with detection and treatment of pre-invasive and early invasive lesions. However, in LMICs, cervical cancer remains amongst the leading causes of cancer mortality. In Zimbabwe, it is the most common malignancy in women, accounting for 31.6% [2] of all cancers in women [2]. Most of these women present with locally advanced disease [3].

Cervical cancer is the most common cancer among women living with human immunodeficiency virus (HIV). HIV positive women are 5 times more likely to develop cervical cancer compared to HIV negative women [4]. The prevalence of HIV in the general population in Zimbabwe has decreased from around 25% to about 12% over the past decade but the rate of HIV infection in cervical cancer patients has remained high.

The standard management of locally advanced cervical cancer (LACC) has remained definitive concurrent chemoradiation (CCRT), which entails external beam radiotherapy (EBRT) with weekly platinum-based chemotherapy and brachytherapy (BT) [5]. With brachytherapy, different dose rates can be used: low, medium and high dose rate i.e. LDR, MDR and HDR respectively. Iridium 192 is the most commonly used isotope for intracavitary HDR brachytherapy worldwide [6]. There is limited data on outcomes from cobalt 60 for high dose brachytherapy in resource-constrained settings. In the study setting, the transition from LDR to use of HDR occurred in 2013 and use of cobalt 60 sources was chosen due to the long half- life. It was considered more economical as source change is required after 5 years compared to Iridium 192 where source change is needed after every 3 months. The less frequent source changes have the advantage of reduced equipment down time and physics support.

Brachytherapy is an essential component of curative therapy for cervical cancer. Addition of brachytherapy leads to improved overall survival by 12% and better local control rates [7, 8] Use of advanced imaging such as CT and MRI for planning has been shown to minimise toxicities and optimize outcomes. High quality brachytherapy can still be delivered, however, even without these advanced imaging modalities [9]. Brachytherapy is necessary to deliver a more conformal boost of dose to the cervix. In bulky tumours it can only be applied when the tumour has regressed from EBRT to a point where insertion of applicators is practical and anatomical distortion from tumour is minimal. Literature suggests that in LACC brachytherapy should be commenced after at least 20Gy of EBRT has been administered [10]. In some instances, however, the tumour may still be too bulky. Brachytherapy may then be administered after EBRT completion, risking prolonged treatment time associated with poorer outcomes if treatment is prolonged beyond 56 days from commencement of EBRT [11, 12]. Generally, 56 days is accepted as the maximum period of total treatment, nevertheless some studies use a cut-off of 63 days [13]. Options for management of disease progression and recurrence for locally advanced cervical cancer such as salvage surgery and chemotherapy are limited and not very effective. This makes it imperative that all aspects of CCRT must be optimized, mainly the brachytherapy boost which is essential to treat definitively LACC.

This study sought to review practices pertaining to brachytherapy in Zimbabwe, including timing with external beam radiotherapy, adverse effects and patient outcomes.

## Methods

A retrospective analysis of records of patients with histologically confirmed cervical cancer treated with CCRT or radiotherapy only (RT) including HDR-BT from 1 January 2013 to 31 December 2014 at Parirenyatwa Group of Hospitals Radiotherapy Centre (PGH-RTC) was carried out. PGH-RTC was the exclusive centre in the country providing radiotherapy services during the study period. The study included all patients with cervical cancer who received EBRT and HDR-BT with or without concurrent chemotherapy. Patients treated with palliative intent were excluded. Brachytherapy was performed according to the departmental protocol using ring and tandem applicators. The ring and tandem applicators have a fixed geometry, enabling reproducible dose distributions, therefore there was no need to plan each subsequent insertion. Planning was done on the first insertion. Dose was prescribed to point A which is 2 cm above the distal end of the lowest source in the cervical canal and 2 cm lateral to the tandem and was chosen to represent the minimum dose to the malignant tissue. Doses to Point B, a point 5 cm lateral to the midline at the same level as point A, which is defined to estimate the dose to the pelvic wall. as well as bladder and rectal point doses were documented. Bladder and rectum were not to exceed 70% of the prescribed dose as per departmental protocol.

Demographic, clinical, pathological and treatment-related data was collected. Treatment details were obtained from the radiation therapy treatment charts and verified in the electronic records on the treatment system namely Aria for EBRT and Bebig HDR plus for brachytherapy.

The data collection tool consisted of three parts. The first section captured patient demographics and co-morbidities. The second section pertained to treatment received by the patient including chemotherapy, scheduling of brachytherapy in relation to external beam therapy and total treatment time. The third section addressed follow up with reference to recurrence and toxicity. Local control was defined as the absence of clinically apparent disease or biopsy-proven recurrence in the treatment field. Overall survival was calculated from the date of the last brachytherapy insertion to the date of last follow up or death. Toxicity was graded using the Common Terminology Criteria for Adverse Event version 3.0.

Biologic effective dose (BED) was calculated using an alpha beta ratio of 10 for the tumour. The EQD2 was obtained by dividing the BED by 1.2. The BED10 for EBRT and for BT were added together to find the total BED10 to point A and the sum of EQD2 for EBRT and BT were obtained for the total EQD2 dose to point A.

The primary outcomes were occurrence of adverse events and local control. Overall survival was the secondary outcome.

Statistical analysis was performed with Epi Info version 3.5.2 statistical package. Data was presented using graphs and . Frequencies and their corresponding percentages were used for univariate analysis. Chi-squared measure of association was used to assess whether there was a statistically significant association between categorical variables. A Chi-squared test for significance was used and a *p*-value of less than 0.05 was considered statistically significant.

Ethical approvals were obtained from the Joint Research and Ethics Committee, (JREC), before commencement of the study.

## Results

Two hundred thirty-eight cervical cancer patients were referred for brachytherapy (BT) between January 2013 and December 2014. [In 9 patients there was difficulty with applicator placement due to cervical stenosis or bulky disease so they were referred for further EBRT to a total dose of 60Gy]. 3 patients did not come for treatment for unknown reasons, so 226 patients were evaluable. The median age of patients was 50 years with a range from 17 to 82 . Most of the patients were HIV negative (59%). Among HIV positive patients, 78% had a CD4 count greater than 200 copies per ml (median 355). Karnofsky performance status (KPS) was 70 and above in 94/96 patients (97.9%), unfortunately KPS was not recorded in all patients. The KPS was however 70 and above in 94/96 (97.9%) of patients whose performance status was recorded (See Table 1). Squamous cell carcinoma was the commonest histology (91%). The cancer stages of patients treated ranged from 1B2 to IVA, with most cancers being in stage IIB (42%). Initial tumour size measurements were available in 212/226 patients, of which 132/212 tumours measured more than 5 cm in greatest dimension (See Table 2).

**Table 1:** Patient Characteristics

Characteristic	Frequency ***n*** = 226 (%)
**Age group in years**	
≤ 30	5 (2)
31–50	111 (49)
51–70	98 (43)
> 70	12 (5)
Median age	50 (Q1 = 42; Q3 = 60)
**Parity**	
0	2 (1)
1–2	48 (21)
3–4	82 (36)
5 +	72 (32)
Not recorded	22 (10)
**HIV status**	
Positive	88 (39)
Negative	133 (59)
Not recorded	5 (2)
**Baseline CD4 count (HIV positive only)**	*N* = 88
< 200	15 (18)
≥ 200	69 (78)
Not recorded	4
**Performance status (KPS)**	
< 70%	2 (1%)
70–90%	94 (42%)
Not recorded	130 (57)

**Table 2:** Disease Characteristics

Characteristic	Frequency n = 226 (%)
**Histology**	
Adenocarcinoma	18 (8)
Adenosquamous	2 (1)
Squamous cell ca.	206 (91)
**Grade (differentiation)**	
Well	5 (2)
Well to moderate	1 (0.5)
Moderate	111 (49)
Moderate to poorly	1 (0.5)
Poorly	100 (44)
Not recorded	8 (4)
**FIGO 2008 stage**	
IB2	18 (8)
IIA	15 (6.5)
IIB	94 (42)
IIIA	18 (8)
III A + B	27 (12)
IIIB	53 (23)
IVA	1 (0.5)
**Initial tumor measurement**	
< 5 cm	62 (28)
5- < 8 cm	118 (52)
8+ cm	14 (6)
Not recorded	32 (14)

### Treatment received

**Fig. 1:**
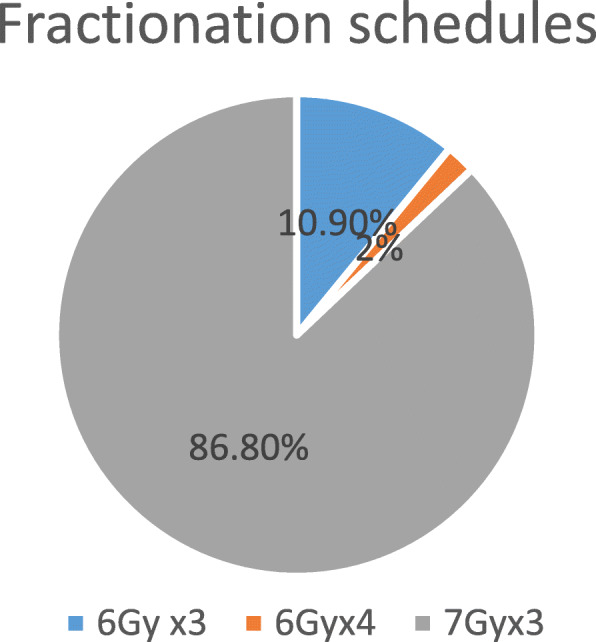
Brachytherapy fractionation regimens

Based on the EBRT total dose received, three HDR-BT fractionation regimens were employed, namely 6Gy × 3, 6Gy × 4 and 7Gy × 3. The 7Gy × 3 regimen was the most employed regimen, accounting for 87%(see Fig. 1). Almost half of patients (52%) commenced brachytherapy whilst still on EBRT. Overall treatment (EBRT and HDR-BT) was completed within 56 days in 70% of patients. The median overall treatment time was 50 days with an interquartile range of 15 days.

### Doses delivered

The dose percentage prescribed to point A which was received by the bladder point during brachytherapy ranged from 10.8 to 82.1% with a median of 43%. The percentage to the rectal point ranged from 11.5 to 79.3 with a median of 55.5% and interquartile range 22.6. Of the 226 patients, only 6 and 8 with bladder and rectal point doses greater than 70% respectively. Point B dose ranged from 18.4 to 36.1% of the dose to point A with a mean of 21.9%. Table 3 shows the range of doses in BED10 and the corresponding EQD2 values and the number of patients receiving those doses.

**Table 3:** Total doses delivered to point A

Total BED10 dose	EQD2	Number of patients
≥90	≥75	177
< 90	74	49

The majority of patients received a total EQD2 less than 90Gy to point A

## Treatment outcomes

### Follow up and survival

**Fig. 2:**
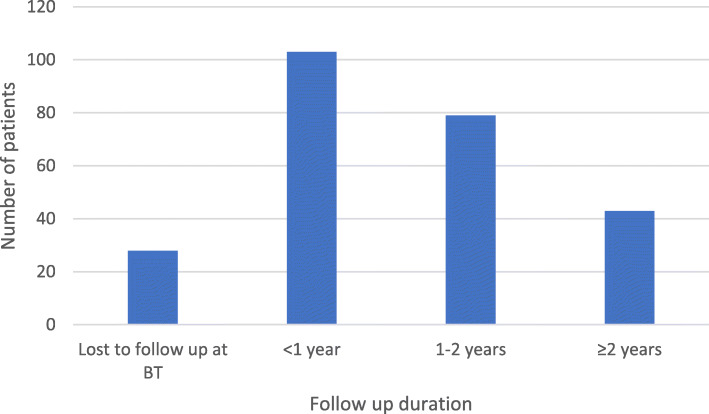
Number of patients reviewed after treatment completion

Fig. 2 illustrates the distribution of patients reviewed for specified periods after treatment completion, 106/226 patients (46%) were reviewed for a period less than 1 year. Twenty-eight patients (12%) were lost to follow-up after treatment completion. These are denoted as lost to follow up at BT in Fig. 2.

### Tumour response

At first review, 6 weeks post treatment, 198/226 (88%) were evaluated. Of 149 (66%) patients had complete clinical response (cCR), 45 (20%) had partial response (PR), 4 had disease progression and 28 (12%) were lost to follow-up (LTFU).

**Fig. 3:**
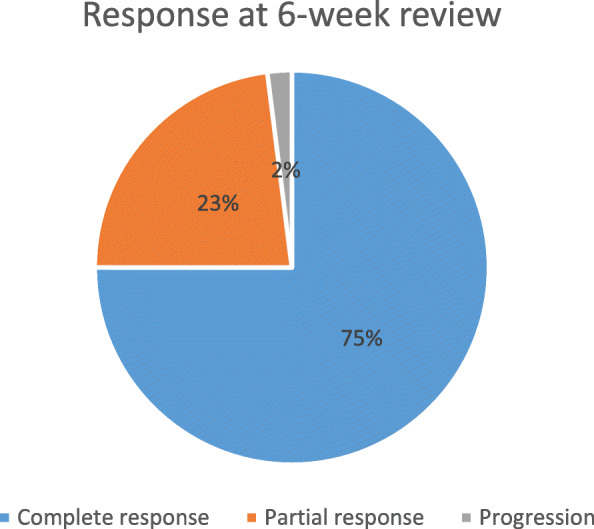
Tumour Response at 6 weeks (*N* = 198)

Figure 3 illustrates the distribution of clinical tumour response in patients reviewedt.

**Fig. 4:**
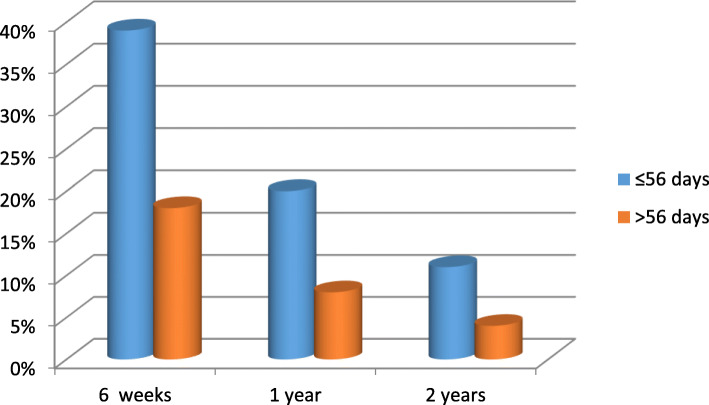
Proportion of patients with no evidence of disease after treatment versus treatment duration

During follow up, complete clinical response was observed in 75, 91 and 95% of patients reviewed at 6-weeks, 1-year and 2-years respectively. The proportion of patients who completed treatment within 56 days overall treatment time and achieved complete clinical response (cCR) were 39, 20 and 11% at 6 weeks, 1-year and 2-years respectively compared to 18, 8 and 4% for those who exceeded 56 days as illustrated in Fig. 4. (*p* = 0.215).

Table 4 shows the distribution of patients with cCR at 6 weeks stratified by clinical stage.

**Table 4:** Proportions of patients with cCR at 6 weeks post-treatment

Stage	No. of patients in Stage Group	No. with cCR at 6 weeks post-treatment
IB2	18	11 (61%)
II	109	74 (68%)
III	98	63 (64%)
IVA	1	1

Disease free survival was 19% at 2 years and 1.8% at 4 years (the time that this study was conducted).

**Fig. 5:**
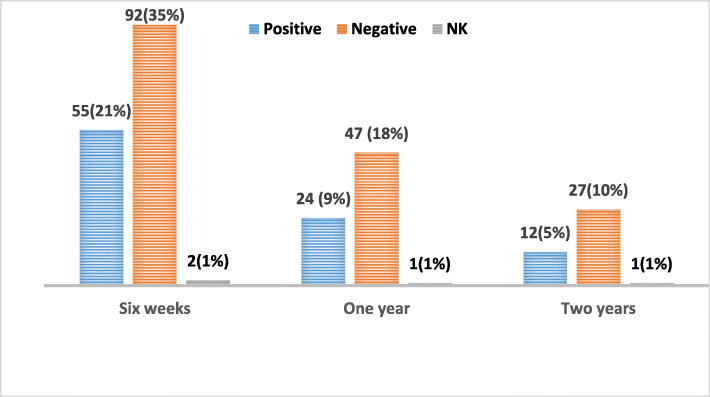
Local Control versus HIV Status

The differences between HIV-infected and un-infected patients with regards to complete clinical response at 6 weeks, 1-year and 2 - years post-treatment was not statistically significant (*p* > 0.05), see Fig. 5.

**Fig. 6:**
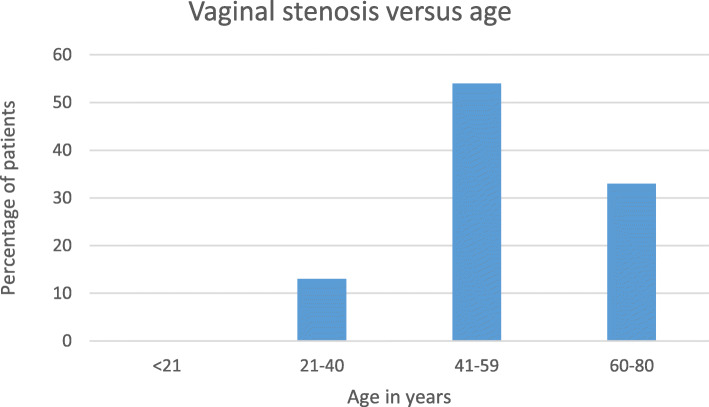
Vaginal stenosis versus age

### HDR-BT fractionation and clinical response

The proportions of patients who achieved cCR at 6 weeks post treatment for the 6Gyx3, 6Gyx4 and 7Gyx3 treatment regimens were 58, 60 and 70% respectively.

### Recurrence

Ten percent of the 226 study participants had documented clinical and/or radiological disease recurrence. Of these, loco-regional, distant and combined recurrences accounted for 26, 65 and 9% of the cases respectively.

Median disease free interval was 14 months (mean 14.5 months) with a range of 4–31 months. All but one of the documented recurrences occurred within the first 2 years’ post-treatment. Table 5 outlines the characteristics in patients who had recurrence.

**Table 5:** Characteristics of Patients Who Recurred (*n* = 23)

Histology	
Adenocarcinoma	3
Poorly differentiated scc	13
Moderately differentiated scc	7
**Stage of disease**	
IIB	13
IIIA	1
IIIB	9
**Concurrent Chemotherapy received**	
Nil	9
< 5 Cycles	12
≥ 5 Cycles	2
**Pattern of Recurrence**	
Loco-regional	6
Distant	15
Local and Distant	2
**Recurrence Site**	
Liver	3
Lung	2
Supraclavicular node	1
Sister Mary Joseph node	1
Liver & lung	2
Lung & Supraclavicular	3
Bone	1
Brain	1
Spine	1

**Table 6:** Summary of documented adverse events

Adverse event	No. of patients		
	Grade 1	Grade 2	Grade 3
**Acute**			
Diarrhoea	10	4	0
Cystitis	14	2	0
**Late**			
Proctitis	11	4	0
Bowel perforation	0	0	0
Recto-vaginal fistula	0	0	2
Bladder perforation	0	0	0
Vesico-vaginal fistula	0	0	4
Vaginal stenosis	38	16	3
Dyspareunia	3	0	0

### Treatment adverse events

The acute toxicities documented were diarrhoea and cystitis, with proctitis, dyspareunia, vaginal stenosis, rectal and vaginal fistulae as the late adverse events reported. Most of the toxicities were grade 1 and 2 (92%) Table 6). The most common adverse effect was vaginal stenosis. Only 3 had severe stenosis where the vaginal lumen was completely closed. The other common toxicities were proctitis, diarrhoea and cystitis.

Of the patients who developed proctitis or cystitis, none had doses to the rectum nor bladder exceeding 70% of point A dose at BT.

There was an increase in incidence of vaginal stenosis with increasing age as shown in Fig. 6 (*p* = 0.00001).

## Discussion

The median age in this study was 50 years, consistent with other studies in literature [14]. Most of the women were of low socio-economic status which tallies with the epidemiology of cervical cancer in other regions and this would result in women not being able to access the necessary treatment [15]. While grand multiparity has been listed as a risk factor for cervical cancer in other literature, only 32% of patients had 5 or more children in this study. Squamous cell carcinoma was the predominant histology (91%) with a significant proportion presented with more advanced disease compared to other histologies [16], consistent with other literature. While there is a role for BT as monotherapy in stages IA to IBI, no patients with these early ICC stages were managed with this treatment modality during the study period. This was expected as the very few early stage ICC cases seen at our centre would have received surgical treatment with hysterectomy already before oncology referral.

The prevalence of HIV infection of 39% in the study population was higher than the documented 13.4% [4] in general population. This was however in keeping with that found in an earlier study on cervical cancer patients done at the Radiotherapy Centre which found a sero-prevalence of 43.5% [17] .

Most of the patients received EBRT to a dose of 45 to 50 Gy to the pelvis as recommended and all the patients completed the prescribed EBRT. Surgical staging of nodal disease was not available and there were no patients treated for para-aortic nodal disease in this cohort. The majority of patients received weekly cisplatin at a dose of 35 mg/m^2^. While initial studies for chemo-radiation used a dose of 40 mg/m^2^ [18], subsequent studies in cervical and head and neck cancer showed similar efficacy with lower toxicity and higher completion rates with doses of 30-35 mg per square metre [19, 20].

Most of the patients in this study commenced HDR-BT in the first 6 weeks enabling treatment completion within the recommended overall treatment time of 8 weeks. A significant proportion (29%) were commenced HDR-BT later after 6 weeks. Twice weekly BT where possible may enable treatment completion within the recommended time frame [10].

Clinical response rates in this study were found not to be associated with the overall treatment time and that’s not in keeping with other studies in literature. This result may be attributed to other adverse features of the patients’ disease such as stage and histology as well as the high lost to follow up rate with resultant missing data impacting on this study’s analysis.

The rectal and bladder doses from the brachytherapy plans were within the accepted limits for most patients. Only a few patients, 6 and 8 out of 226 had bladder and rectal point doses greater than 70% respectively. Patients who developed cystitis and proctitis received doses less than 70%. However, exact total doses to bladder and rectum could not be determined as the doses to these from EBRT were unknown even though it can be estimated that antero-posterior, postero-anterior fields (APPA) would give approximately 100% of the dose to these structures.

The total BED delivered to point A ranged from 81.9 to 100.5 which equates to an EQD2 of 68.3 to 83.8. While an EQD2 of 85-90Gy is recommended for tumours greater than 5 cm, some studies showed similar outcomes for local failure rates by stage to other studies that used the American Brachytherapy Society (ABS) recommended doses even though total doses to point A were 10-15Gy lower than the LDR equivalent doses recommended in the (ABS) guidelines [21, 22]. Furthermore, a literature analysis by Petereit found no correlation between point A BED and local control nor toxicity, although it suggested that a dose below 46-79Gy10 would not be tumoricidal [23].

A high loss to follow rate at 2 years of 81% was noted in this study. The loss to follow up may be due to financial constraints, patient deterioration and demise or a lack of understanding about the importance of follow up.

The complete clinical response rate of 75% at 6 weeks was lower than in other studies where the rates were above 90% for patients who receive EBRT plus BT. The response rate in this study, however was comparable to the 73% in patients who received EBRT only from a study by Karlsson et al. [8]. Improvement in conformal EBRT technology for example intensity modulated radiation therapy (IMRT) and volumetric modulated therapy (VMAT), where available, enables planning of dose boost to positive parametria or lymph nodes which can impact the clinical response rate. Interstitial brachytherapy would be useful in treatment of positive parametrium if this were available at our centre.

In this study, the cCR rates for the 3 schedules were similar i.e. 60% for 6Gyx3, 60% for 6Gyx4, and 70% for 7Gyx3 suggesting no difference in outcomes with the different regimens. This is consistent with findings from other studies were no optimal schedule was identified.

Of the 23 recurrences documented in this study, the majority (63%) were distant and this is in keeping with findings from other authors [24, 25].

Overall survival data could not be fully established with the high lost to follow up rate. If assuming that the 43 patients who attended review 2 years after treatment were the only living patients, that would give a low 2-year overall survival of 19%. A study by Maranga et al. in Kenya showed a 2-year survival of less than 20% by Kaplan Meier projection in a group of women treated with EBRT, BT and adjuvant chemotherapy.

Khalil et al. showed an overall survival rate of 68% at 2 years and a local control rate of 71%. Grade 3 or 4 toxicity was at 20% in a study of patients with locally advanced cervical cancer in Morocco [26]. They concluded that outcomes of locally advanced cervical cancer remained poor in spite of the treatment available.

There was some variation in grading of adverse effects, with some using pluses and others writing mild or moderate. Most of the toxicities were Grade 1 and 2 in severity i.e. mild to moderate. Only vaginal stenosis and fistulae had grade 3 events. The occurrence of fistulae may be a result of radiotherapy, but may also occur as a result of advanced disease. No bowel or bladder perforation was reported. While these adverse effects (bladder and bowel perforation) are rare, there is a possibility that patients who do perforate may present as acute abdomen to other medical specialists and not report to oncologists. There was a statistically significant increase in occurrence of vaginal stenosis with increasing age which is consistent with some studies (*p* = .00001) [27, 28].

The limitation of this study is its retrospective nature. Some information was missing from the records and also there was no uniformity in classification of variables. The high lost to follow up rate impacted on the analysis of patient’s outcomes.

## Conclusion

Brachytherapy plays an important role in the management of cervical cancer. Cobalt 60 brachytherapy resulted in response comparable to Iridium 92. Similar outcomes were observed with the different brachytherapy regimens used. Most patients commenced HDR-BT whilst on EBRT and completed treatment within the recommended 56 days’ overall treatment time with a high complete clinical response rate at 6 weeks post-treatment as expected. There was however, a high lost to follow up rate at 1 and 2 years. Brachytherapy using high dose rate cobalt 60 sources in addition to external beam radiotherapy provided safe, well tolerated treatment with low numbers of grade 3 adverse effects. Prospective studies are recommended in this patient population to improve long term outcomes.

## Data Availability

The datasets analysed during the current study available from the corresponding author on reasonable request.
