# Correction to: High dose rate intra-cavitary brachytherapy with cobalt 60 source for locally advanced cervical cancer: the Zimbabwean experience

**DOI:** 10.1186/s13027-021-00348-5

**Published:** 2021-02-11

**Authors:** Shirley Chibonda, Ntokozo Ndlovu, Nomsa Tsikai, Lameck Munangaidzwa, Sandra Ndarukwa, Albert Nyamhunga, Tinashe Mazhindu

**Affiliations:** 1 Parirenyatwa Hospital Radiotherapy and Oncology Centre, Harare, Zimbabwe; 2 Department of Oncology, University of Zimbabwe Faculty of Medicine and Health Sciences, Harare, Zimbabwe; 3 Department of Statistics, National AIDS Council of Zimbabwe, Harare, Zimbabwe; 4 Department of Oncology, Sally Mugabe Central Hospital, Harare, Zimbabwe

**Correction to: Infect Agents Cancer 16, 1 (2021)**

**https://doi.org/10.1186/s13027-020-00340-5**

The original publication [1] of this article was published with an incorrect title. In this correction article the incorrect and correct title are shown, the original publication has been updated.

**Incorrect**

- Working title: high dose rate intra-cavitary brachytherapy with cobalt 60 source for locally advanced cervical cancer: the Zimbabwean experience

**Correct**

- High dose rate intra-cavitary brachytherapy with cobalt 60 source for locally advanced cervical cancer: the Zimbabwean experience

## References

[1] Chibonda S, Ndlovu N, Tsikai N (2021). Working title: high dose rate intra-cavitary brachytherapy with cobalt 60 source for locally advanced cervical cancer: the Zimbabwean experience. Infect Agents Cancer.

